# From Emotional (Dys)Regulation to Internet Addiction: A Mediation Model of Problematic Social Media Use among Italian Young Adults

**DOI:** 10.3390/jcm11010188

**Published:** 2021-12-30

**Authors:** Alessandro Quaglieri, Silvia Biondi, Paolo Roma, Manuel Varchetta, Angelo Fraschetti, Jessica Burrai, Giulia Lausi, Manuel Martí-Vilar, Francisco González-Sala, Alberto Di Domenico, Anna Maria Giannini, Emanuela Mari

**Affiliations:** 1 Department of Psychology, Sapienza University of Rome, Via dei Marsi 78, 00185 Rome, Italy; alessandro.quaglieri@uniroma1.it (A.Q.); manuelvarchetta@gmail.com (M.V.); angelo.fraschetti@uniroma1.it (A.F.); jessica.burrai@uniroma1.it (J.B.); giulia.lausi@uniroma1.it (G.L.); annamaria.giannini@uniroma1.it (A.M.G.); 2 Department of Human Neuroscience, Sapienza University of Rome, Piazzale Aldo Moro 5, 00185 Rome, Italy; silvia.biondi@uniroma1.it (S.B.); paolo.roma@uniroma1.it (P.R.); 3 Departamento de Psicología Básica, Facultad de Psicología y Logopedia, Universidad de Valencia, Av. Blasco Ibáñez, 21, 46010 Valencia, Spain; manuel.marti-vilar@uv.es; 4 Departamento de Psicología Evolutiva y de la Educación, Facultad de Psicología y Logopedia, Universidad de Valencia, Av. Blasco Ibáñez, 21, 46010 Valencia, Spain; francisco.gonzalez-sala@uv.es; 5 Department of Psychological, Health and Territorial Sciences, University “G. d’Annunzio” of Chieti-Pescara, Via dei Vestini 31, 66100 Chieti, Italy; alberto.didomenico@unich.it

**Keywords:** internet addiction, fear of missing out, social media addiction, mediation, behavioral addiction, personality traits, risk factors, emotion, disorder

## Abstract

Internet addiction (IA) has mostly been investigated with the fear of missing out and difficulties in emotional regulation. The present study examined the link between IA and variables related to problematic social media use (i.e., fear of missing out, social media addiction), together with emotional (dys)regulation and personality traits, providing new insights and an integrated assessment of IA. In total, 397 participants, aged 18–35 years (*M* = 22.00; *SD* = 3.83), were administered a set of questionnaires pertaining to IA, problematic social media use, emotional (dys)regulation, and personality traits. Pearson’s correlations showed significant associations between IA and the investigated variables, and the tested mediation model highlighted the crucial role played by emotional (dys)regulation in the fear of missing out and problematic use of social networks. Overall, the findings provide support for a new integrated model for understanding the features, predictors, and risk factors of IA.

## 1. Introduction

Internet addiction (IA), also known as problematic internet use, has been defined as problematic and compulsive use of the internet, resulting in significant impairments to daily life functioning. IA is closely linked to computer addiction, which may otherwise be referred to as online addiction and technological addiction. More in detail, IA is an addictive disorder characterized by intensive and obsessive use of the internet, including social networking, video, and online gaming sites [1,2].

In particular, social network overuse can be considered as one form of IA, where individuals exhibit a compulsion to use social media [3,4] leading to the possible onset of pathological behaviors. Individuals with social media addiction exhibit an uncontrollable urge to “log in” and to use social media [5]. Furthermore, studies on adolescent populations found out correlations between frequency in the use of social networks (SNs) and diagnostic criteria for IA [6,7,8].

As such, it encapsulates a wide range of psychopathological behaviors (e.g., online gambling addiction, online gaming addiction, communication addiction disorder), whose common matrix seems to be impulse dysregulation [9,10,11] and difficulties in managing emotional states [12,13,14,15].

Dysregulated emotional responses may foster addictive behavior as a means of coping with high levels of stress. In this vein, individuals with a propensity towards emotional distress have been found to be more likely to engage in substance use. Specifically, studies of opioid [16,17], cocaine [18,19], methamphetamine [20,21], alcohol [22,23], and tobacco [24,25] misuse have shown a close link between addiction and emotional dysregulation. Furthermore, the role of emotional (dys)regulation and alexithymia, often conceptualized as emotional regulation disorder [26], has also been investigated in non-substance–related addictions, including gambling disorder [27,28], IA [29,30], gaming addiction [28,31], and problematic internet use [32]. Again, emotional (dys)regulation has been found to strongly correlate with the severity of these behavioral addictions [28]. Studies have also shown that individuals with both emotional (dys)regulation and alexithymia experience difficulties developing healthy relationships, due to their problematic management of emotional states, and consequent mood disorders. Such individuals may consider the internet an arena in which they can achieve greater self-control and engage in better communication with others [30].

Despite this empirical evidence of the role of emotional dysregulation in addictive behavior, many questions still remain. In particular, it is not yet known whether emotional dysregulation is a cause, correlation, or consequence of addictive behavior.

### 1.1. Internet Addiction and Emotional (Dys) Regulation

IA is not currently listed as a formal disorder by the main diagnostic classifications, such as the Diagnostic and Statistical Manual of Mental Disorders—fifth edition (DSM-5) [33] and the International Classification of Diseases—11th Revision (ICD-11) [34]. However, the inclusion of one of its phenomenological manifestations—internet gaming addiction disorder—has been proposed in the DSM-5, while it is described as a manifestation of the Gaming Disorder (6C51) in the ICD-11. Young [35] was the first to outline specific diagnostic criteria for IA, based on the DSM-IV diagnostic criteria [36] for pathological gambling, with which IA shares symptomatic overlap; drawing on this criteria, she constructed a diagnostic questionnaire (i.e., Young’s Internet Addiction Test; IAT) [35]. Research using the IAT has shown that IA has characteristics and symptoms comparable to those of gambling disorder (e.g., preoccupation, tolerance, withdrawal, failure to control, impaired decision making) and comorbidity with depression and anxiety [37,38,39], obsessive-compulsive symptoms [40,41,42,43], attention deficit and hyperactivity disorder [44], and hostility–aggression behaviors [45,46]. Nevertheless, IA is not classified as a specific psychiatric disorder, but a psychological symptom that may arise in various psychopathological frameworks. Therefore, it may be more useful to identify the individual and environmental factors that predict high internet use than to diagnose IA as a specific addiction or primary disorder.

The internet enables users to connect with others, discover new worlds, acquire new knowledge, and create positive emotions (e.g., happiness, fun, satisfaction). Therefore, IA might reflect an individual’s attempt to cope with and escape from negative emotions in everyday life and promote more positive affect. However, the positive emotional reinforcement that may arise from use of the internet can easily produce an imbalance, leading to abuse or overuse [14]. Indeed, a study by Longstreet et al. [14] showed that IA increased negative and decreased positive emotional states; this suggests that the positive emotional reinforcement that can stem from use of the internet may encourage continued internet use.

Furthermore, deficiencies in the ability to effectively identify and describe emotions (i.e., alexithymia) has been shown to significantly predict IA. For instance, in a sample of adolescents, IA was strongly correlated with a reduced ability to understand emotional reactions, control impulsive behavior in response to negative emotional experiences, and use effective emotional regulation strategies [47]. The use of IA as an escape behavior and manner of coping with emotional difficulties and stressful events has also been confirmed in a sample of young adults: Pettorruso et al. [48] showed that the presence of emotional problems, especially in young adults with low novelty-seeking, predicted the development of problematic social interaction (i.e., problematic use of the Internet), leading to detachment from reality.

### 1.2. Social Media Addiction and Fear of Missing Out 

Fear of missing out (i.e., FoMO) has been defined as an individual’s fear that other people are having fun without them [49]. Wegmann et al. [50] suggested that FoMO is not a unitary phenomenon, but a complex construct reflecting both a personal predisposition and a specific cognition regarding the fear of missing out on a particular event. Although the theoretical construct of FoMO does not explicitly refer to a specific context (i.e., online, offline), most studies have applied the construct to the online context [49,51,52]. Furthermore, FoMO has been found to be associated with a variety of psychopathological symptoms, including social isolation, depression, and anxiety [53,54,55,56,57]. 

Several studies have linked FoMO to the overuse of SNs [58,59,60,61], which may be considered ideal for satisfying the “desire to stay continuously connected to what others are doing” [49,62]. FoMO-related overuse of SNs [49,63] may manifest in the problematic use of smartphones [64], resulting in a vicious circle [65] whereby frequent and compulsive checking of social media on one’s smartphone may lead individuals to actively seek out events they have missed [57,66]. 

Excessive use of SNs may also lead to social media addiction—an uncontrollable and compulsive behavior [40,67,68] that has been shown to be associated with emotional, relational, well-being, and performance problems [69,70,71,72]. 

Although FoMO has been identified as potentially responsible for problematic use of social networks, it should come as no surprise that compulsive users of social networks may experience FoMO as a result. Individuals engaging in higher levels of social network use seem to be more likely to experience online vulnerability and exhibit higher levels of FoMO [73]. One possible reason could be the excessive social surveillance that SNs provide; indeed, prior to both the advent and spread of SNs, individuals were less likely to be aware of the activities of “others”. The continuous exposure to this information could lead individuals to believe that “others” are having a better life and that one is missing out on something. 

The continuous need to stay in touch offered by social media may, therefore, exacerbate the FoMO in accordance with the reinforcement spiral model [74], which emphasizes that the use of particular media content may reinforce those needs that led to its initial use. This could place the individual into a spiral of behavior in which it will be difficult to satisfy the sense of control and social belonging.

### 1.3. Personality Traits

Research has demonstrated a strong relationship between personality and IA, as well as behavioral addictions, more generally [75,76,77,78]. In this context, several studies have investigated the influence of the “Big Five” personality traits (i.e., extroversion, agreeableness, conscientiousness, neuroticism, and openness to new experiences) [79] on internet behavior. Such studies have found that conscientiousness—defined as the propensity to “follow socially prescribed norms for impulse control, to be goal-oriented, to plan and to be able to delay gratification and to follow norms and rules” [80]—is negatively associated with excessive social media use [81] and IA [82]. In a similar vein, Andreassen et al. [75] found that conscientiousness was a protective factor for unproductive behavioral addictions (e.g., addiction to Facebook, video games, or the internet) and a risk factor for positive or productive behavioral addictions (e.g., an addiction to studying). On the other hand, neuroticism has been found to predict both social media use and IA, especially in studies using age as a covariate [53]. Neuroticism, or the “tendency to experience negative emotional states, characterized by a tendency to worry and be anxious” [83,84], has been found to be associated with frequent and unbalanced negative affect in response to stressful circumstances [85]. In addition, Ehrenberg et al. [86] found that individuals with high neuroticism showed stronger mobile phone addictive tendencies and a preference for online communication.

### 1.4. Study Aim and Hypotheses 

The present study aimed at investigating the relationships between IA and variables identified in the literature as related to this construct (i.e., social media addiction, FoMO, difficulties in emotional regulation, personality traits). Specifically, the hypotheses were as follows:

**Hypothesis** **1** **(H1).** *The following correlations identified in the literature would be verified:*

*a.*  *Positive correlation between IA and variables related to problematic social media use;*
*b.*  *Positive correlation between IA and emotional (dys)regulation; and*
*c.*  *Correlation between IA and personality traits; in particular, a positive correlation with neuroticism and a negative correlation with conscientiousness.*

**Hypothesis** **2** **(H2).** *There would be a direct positive effect of emotional (dys)regulation (the independent variable) on IA (the dependent variable).*

**Figure 1:**
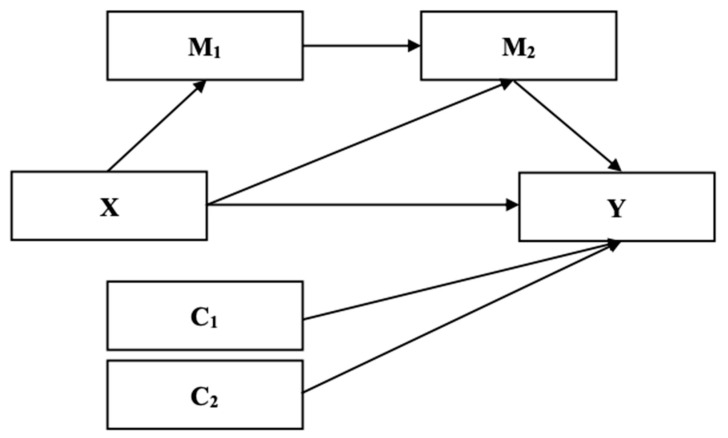
Proposed mediation model (Model 6). Note. X = independent variable; Y = dependent variable; M_1_ = mediator 1; M_2_ = mediator 2; C_1_ = Covariate 1; C_2_ = Covariate 2.

**Hypothesis** **3** **(H3).** *There would be an effect of the independent variable on the proposed mediator (FoMO and social media addiction), and an effect of the proposed mediator on the dependent variable; and an effective mediation model could be established including neuroticism and conscientiousness as covariates (Figure 1).*

## 2. Materials and Methods

### 2.1. Participants

The sample was comprised of 397 Italian young adults (122 male, 274 female, 1 other). Ages ranged from 18 to 35 years, with an average age of 22.00 years (*SD* = 3.83). With respect to education, 1.8% had a primary or middle school diploma, 73.6% had a high school diploma, 16.6% had a bachelor’s degree, 7.1% had a master’s degree, and 1.0% had a postgraduate degree. Regarding occupation, a vast majority of participants were students (80.9%), 0.3% worked in law enforcement, 2.3% were conceptual employees, 1.3% were executive employees, 1.0% were managers, 0.8% were teachers, 1.5% were workers, 2.5% were practitioners, 1.8% were healthcare professionals, and 7.8% were “other”.

### 2.2. Measures

#### 2.2.1. Internet Addiction Test

The Internet Addiction Test (IAT) [35] is a widely used instrument for assessing IA. It consists of 20 items (e.g., “How often do you find that you stay online longer than you intended?”, “How often do you form new relationships with fellow Online users?”) that are rated on a 5-point Likert scale ranging from 1 (very rarely) to 5 (very frequently). Higher scores indicate higher levels of IA (0–30 = normal range; 31–49 = mildly addicted; 50–79 = moderately addicted; and 80–100 = severely addicted) [87]. The present study applied the Italian version of the IAT, developed by Fioravanti and Casale [88]. Cronbach’s alpha was 0.85.

#### 2.2.2. Bergen Social Media Addiction Scale

The Bergen Social Media Addiction Scale (BSMAS) [40] is a self-report questionnaire pertaining to social media addiction. It is based on the six dimensions proposed by Griffiths [89] (i.e., salience, mood, modification, tolerance, withdrawal conflict, relapse) and consists of six items (e.g., “How often during the last year have you tried to cut down on the use of social media without success?”) that are rated on a 5-point Likert scale ranging from 1 (very rarely) to 5 (very often). Higher scores indicate a greater risk of developing social media addiction, with the score of 24 recently indicated as the best cut-off score for a possible clinical diagnosis [90]. The present study applied the Italian version of the test, developed by Monacis et al. [91]. Cronbach’s alpha was 0.78.

#### 2.2.3. Fear of Missing Out Scale 

The Fear of Missing Out Scale (FoMOs) [49] is a 10-item (e.g., “I get anxious when I do not know what my friends are up to”) self-report questionnaire that measures respondents’ experiences of a pervasive apprehension that “others” are engaged in positive activities and relationships without them. Items are rated on a 5-point Likert scale ranging from 1 (not at all true of me) to 5 (extremely true of me). The present study applied the Italian version of the scale, developed by Casale and Fioravanti [92]. Cronbach’s alpha was 0.83.

#### 2.2.4. Difficulties in Emotion Regulation Scale

The Difficulties in Emotion Regulation Scale (DERS) [93] is a 36-item self-report questionnaire (e.g., “I pay attention to how I feel”, “When I’m upset, I become irritated at myself for feeling that way”) that measures six facets of difficulty regulating emotions (i.e., non-acceptance, goal-directed behavior, impulse control, limited access to effective emotional regulation strategies, lack of emotional awareness, lack of emotional clarity). The present study applied the Italian short version of the scale, proposed by Lausi et al. [94], composed of 20 items rated on a 5-point Likert scale ranging from 1 (almost never) to 5 (almost always). Higher scores indicate greater difficulties in emotional regulation. Cronbach’s alpha was 0.88 for the total scale, 0.89 for non-acceptance, 0.91 for goal-directed behavior, 0.91 for impulse control, 0.86 for lack of emotional clarity, and 0.82 for lack of emotional awareness.

#### 2.2.5. Big Five Inventory-10

The Big Five Inventory-10 (BFI-10) [95] is a self-report questionnaire that evaluates five personality dimensions (i.e., Extroversion, Agreeableness, Conscientiousness, Neuroticism, Openness to Experience). Items (e.g., “I see myself as a person that is reserved”, “I see myself as a person that tends to find fault with others”) are rated on a 5-point Likert scale ranging from 1 (strongly disagree) to 5 (strongly agree). The present study applied the Italian version of the scale, developed by Guido et al. [96]. The reliability of each personality dimension was tested using Spearman–Brown coefficients, indicating: 0.69 for Extroversion, 0.39 for Agreeableness, 0.69 for Conscientiousness, 0.42 for Neuroticism, and 0.71 for Openness to Experience.

### 2.3. Procedure

Participants were recruited online, and they voluntarily and anonymously responded to the survey, which they accessed through a designated link (Qualtrics Online Platform). Participants indicated their informed consent prior to beginning the survey, and they were free to interrupt or quit the survey at any point, without providing an explanation for doing so. Expedited ethics approval was obtained from the Institutional Board of the Department of Psychology, Faculty of Medicine and Psychology, “Sapienza” University of Rome (IRB 2193/2020), in accordance with the principles embodied in the Declaration of Helsinki.

### 2.4. Data Analysis

Pearson’s correlation coefficients (*r*) were calculated to study the associations between scores on the IAT, BSMAS, FoMOs, DERS, and BFI-10 Neuroticism and Conscientiousness (sub)scales. The mediation model was run using PROCESS version 3.5, as developed by Preacher and Hayes for SPSS, version 26 (IBM, Armonk, NY). PROCESS estimates indirect effects (i.e., mediation) and conditional indirect effects (i.e., moderated mediation) using bootstrap confidence intervals. In the present study, the bias-corrected 95% confidence interval (CI) was calculated using 5000 bootstrapping resamples. Effects were considered significant when the resulting confidence interval did not contain 0. A serial mediation model (Model 6) was tested using PROCESS Model Templates, in order to explore whether the association between difficulties in emotional regulation (i.e., DERS) and IA (i.e., IAT) were mediated by social media addiction (i.e., BSMAS) and FoMO (i.e., FoMOs), using BFI-10 Neuroticism and Conscientiousness subscale scores as covariates.

## 3. Results

Table 1 presents the descriptive statistics for the main study measures.

Pearson’s correlation analysis (see Table 2) showed that the IAT score was significantly and positively correlated with all variables (i.e., BSMAS, FoMOs, DERS, BFI-10 Neuroticism scores), and negatively correlated with the BFI-10 Conscientiousness score. In more detail, the IAT score showed a strong positive correlation with the BSMAS score; a moderate positive correlation with the FoMOs, DERS, and BFI-10 Neuroticism scores; and a weak negative correlation with the BFI-10 Conscientiousness score.

Furthermore, the correlation analysis highlighted a moderate positive correlation between all variables (i.e., BSMAS, FoMOs, DERS, and BFI-10 Neuroticism scores), except for the BFI-10 Conscientiousness score, which showed a weak negative correlation with all the aforementioned variables.

Subsequently, we tested the prediction concerning the link between difficulties in emotional regulation (i.e., DERS), IA (i.e., IAT), media addiction (i.e., BSMAS), and FoMO (i.e., FoMOs). The total effect of the DERS score on the IAT score (*B* = 0.230 [*SE*(HC0) = 0.04] *p* < 0.001 [CI = 0.1522, 0.3071]) was significant. Furthermore, as shown in Table 3, the indirect effect of the DERS score on the IAT score via the BSMAS and FoMOs scores was positive and significant (*B* = 0.161), and the bootstrapped 95% CI did not include 0 [0.0990, 0.2250].

**Figure 2:**
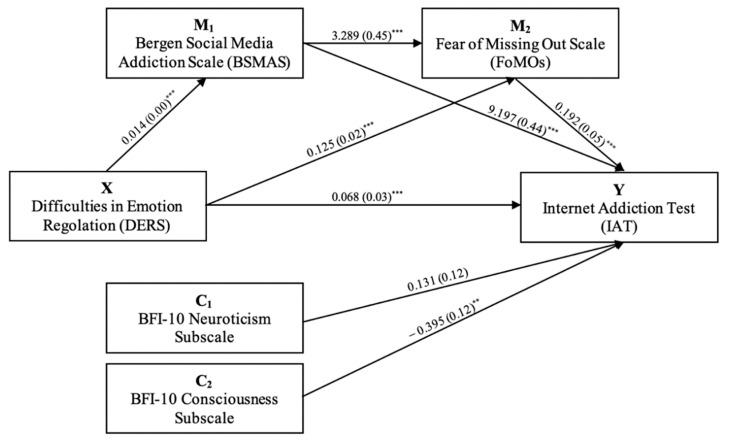
Mediation model in which the mediators were considered sequentially. Numbers represent standardized coefficients. Numbers within parentheses represent standardized errors. Note. X = independent variable; Y = dependent variable; M_1_ = mediator 1; M_2_ = mediator 2; C_1_ = Covariate 1; C_2_ = Covariate 2; ** = *p* < 0.01; *** = *p* < 0.001.

In the final model, the BFI-10 Neuroticism and Conscientiousness scores (as covariates) were significantly positively (*B* = 0.807) and negatively (*B* = −0.496) associated with the IAT score, respectively (Figure 2).

The final mediation model explained 25% of the variance in IAT. 

## 4. Discussion

The present study examined the connections between IA and variables related to problematic social media use (e.g., social media addiction, FoMO), as well as the connections between IA and emotional (dys)regulation. Despite the considerable literature on this topic, the research aimed at contributing a deeper analysis of the role of emotional (dys)regulation and the interaction between variables related to internet use in the development of IA.

The results were largely consistent with the predictions. Specifically, the first hypothesis (H1) was confirmed: IA was found to correlate with all the investigated variables, demonstrating a strong and positive relation to social media addiction, FoMO, emotional (dys)regulation, and neuroticism; and a weak and negative relation to conscientiousness. Moreover, all variables were found to significantly relate to each other, supporting the frame of a vicious circle whereby underlying emotional (dys)regulation exacerbates problematic social media use and the pervasive apprehension that others are engaged in exclusive positive activities, resulting in severe IA. These findings seem to align with the pathways hypothesis pertaining to a specific group of behavioral dependents (i.e., problematic and pathological gamblers). This hypothesis was first proposed by Blaszczynski and Nower [97], and subsequently elaborated by Ledgerwood and Petry [98] and Valleur et al. [99]. In their studies, these researchers found that individuals showed poor emotional coping skills and experienced more depression, anxiety, and emotional dysregulation prior to the onset of their addiction, which likely emerged as an attempt to ameliorate these negative symptoms. These findings seem to be in line with the “compensatory internet use” model developed by Kardefelt-Winther [100] in which IA was proposed as a coping strategy and presented from a perspective of compensation instead of compulsive behavior. In particular, the negative life situations experienced by the individual may find, in online activities, a way of escaping from reality to alleviate or ameliorate their dysphoric mood problems. The effectiveness of this model appears to be stronger with specific motivations (e.g., life difficulties, negative affect states, higher levels of stress) that precede problematic outcomes.

Furthermore, impulsivity, together with emotional and personality variables, were found to be associated with addictive behaviors in a sample of adolescents, resulting as vulnerable factors for the development of psychopathological diseases in adults [15]. The presence of impulsiveness alongside a personality characterized by high neuroticism seems to predispose individuals to compulsive behavior. As Griffiths [3] showed, excessive use of the internet is often linked to online social contact (e.g., use of chat rooms). More compulsive behavior may consolidate this relationship, potentially resulting in social media addiction. Indeed, social media addiction is one of the most common forms of IA, involving the compulsive use of social media [4,67]. Additionally, Oberst et al. [57] revealed that immersion in SNs can increase one’s sense of belonging in a very positive way, particularly among socially vulnerable populations. At the same time, increased use of SNs can also exacerbate FoMO. In line with the first hypothesis, the present study found a positive correlation between use of SNs and FoMO, suggesting that participants may have used SNs to fulfil a need for connection or belonging (even through online networks), potentially developing addictive behavior in this respect.

Lastly, conscientiousness emerged as a protective factor against behavioral addictions defined as “unproductive” (e.g., addiction to Facebook, video games, or the internet), while neuroticism emerged as a general risk factor for the development of psychopathology, as underlined in the literature [75,82,101,102].

The second hypothesis (H2) was also confirmed: the mediation model showed a direct effect of emotional (dys)regulation on IA, suggesting that emotional regulation may play an important role in the development of IA. Indeed, studies have shown that individuals with IA—especially adolescents—have greater difficulties with emotional regulation, resulting from excessive suppression and poor cognitive reappraisal [47,103,104]. The excessive suppression of negative emotional experiences involved in emotional dysregulation may strengthen its correlation with IA. In accordance with this, the present study found that deficits in emotional regulation resulted in a stronger correlation between negative emotions and IA. Overall, the findings suggest that difficulties managing negative emotions could predict problematic use of the internet and its features, in line with the study of Amendola et al. [105], which found that dimensions of “non-acceptance” and “goals” predicted problematic Internet use.

Finally, the third hypothesis (H3) was also confirmed: the final mediation model showed the influence of emotional (dys)regulation on FoMO and problematic use of social networks, with a consequent effect on IA. While several studies have investigated the relationship between FoMO and problematic smartphone use [64,106] and social media addiction [59,61], little is known about the relationship between FoMO and emotional (dys)regulation. The present results underline a significant effect of emotional (dys)regulation on FoMO, bringing to light a direct relationship between the constructs, whereby an increase in one may predict an increase in the other. The same direct relationship was observed between emotional (dys)regulation and social media addiction, further highlighting the crucial role played by the ability to manage emotions in the development of behavioral addictions. These findings are aligned with the results of previous studies, which have found a strong association between difficulties regulating emotions and problematic use of SNs [107]. In addition, the significant relationship between these three variables had an overall effect on IA, confirming the direct effect of emotional (dys)regulation, FoMO, and social media addiction on the possible development of pathological behavior related to use of the internet.

## 5. Conclusions

The results of the present study contribute new insights to the literature on the role of emotional (dys)regulation in the development of pathological Internet behaviors. Plausible mediation pathways were tested, based on theoretical models and research suggesting that emotional (dys)regulation could mediate the development of addictive behaviors. Overall, the results suggest that emotional (dys)regulation could be conceptualized as an independent factor and direct predictor of both IA and variables related to problematic social media use. Further investigations should aim at identifying the specific personality traits and interventions that are effective at decreasing the maladaptive aspects of IA while increasing or enhancing its adaptive aspects.

In summary, mediation was revealed between emotional (dys)regulation, IA, and variables related to problematic internet use. Further research should test models that will further our understanding of the relationship between emotional regulation and its adaptive and maladaptive effects. The results of such investigations may facilitate the development of more suitable approaches to assess and treat IA and evaluate outcomes associated with emotional regulation. Research should also seek to replicate the current findings and further investigate which aspects may influence or be influenced by emotional (dys)regulation (e.g., gender differences), in a variety of populations (e.g., college students vs. non-college-educated adults, young vs. older adults, clinical vs. non-clinical samples). Finally, the causal conjectures of the present study design should be examined and verified via longitudinal or experimental research.

Nevertheless, some limitations of the present study must be considered. First, we were unable to draw causal conclusions or establish the direction of effects, due to the cross-sectional research design. For this reason, it cannot be ruled out that, in some cases, emotional (dys)regulation and the presence of IA may have exerted a mutual influence, or emotional dysregulation may have evolved after the onset of IA. Furthermore, the present study used only self-report measures to assess IA and variables related to problematic social media use; these measures were unable to provide diagnoses, thereby limiting the clinical implications of the results. Furthermore, the study sample may have been affected by selection bias, since the questionnaires were more accessible to certain groups of individuals. Finally, participants’ psychiatric history was not collected, though this data might have influenced the interpretation of the results.

## Figures and Tables

**Table 1:** Descriptive statistics for the IAT, BSMAS, FoMOs, DERS, and BFI-10 Neuroticism and Conscientiousness measures.

	*M* (*SD*) *n* = 397	Median *n* = 397
IAT Total	43.47 (9.40)	43.00
BSMAS	12.06 (4.04)	12.00
FoMOs	25.08 (6.48)	25.00
DERS	51.74 (12.13)	52.00
DERS_NA	12.47 (4.98)	12.00
DERS_AW	7.40 (3.09)	7.00
DERS_CL	8.47 (2.80)	8.00
DERS_IM	9.49 (3.88)	9.00
DERS_GO	13.91 (3.95)	14.00
BFI-10		
Neuroticism subscale	10.23 (2.65)	10.00
Conscientiousness subscale	12.03 (2.45)	13.00

Note. IAT: Internet Addiction Test; BSMAS: Bergen Social Media Addiction Scale; FoMOs: Fear of Missing Out Scale; DERS: Difficulties in Emotion Regulation Scale; DERS_NA = Nonacceptance dimension of Difficulties in Emotion Regulation Scale; DERS_AW = Awareness dimension of Difficulties in Emotion Regulation Scale; DERS_CL = Clarity dimension of Difficulties in Emotion Regulation Scale; DERS_IM = Impulse dimension of Difficulties in Emotion Regulation Scale; DERS_GO = Goals dimension of Difficulties in Emotion Regulation Scale; BFI-10: Big Five Inventory-10.

**Table 2:** Correlation coefficients (Pearson’s *r*) between the IAT, BSMAS, FoMOs, DERS, and BFI-10 Neuroticism and Conscientiousness (*n* = 397) scores.

	IAT	BSMAS	FoMOs	DERS	BFI-10 Neuroticism	BFI-10 Conscientiousness
IAT	-	0.786 **	0.526 **	0.444 **	0.396 **	−0.250 **
BSMAS	0.786 **	-	0.492 **	0.380 **	0.371 **	−0.146 **
FoMOs	0.526 **	0.492 **	-	0.447 **	0.407 **	−0.152 **
DERS	0.444 **	0.380 **	0.447 **	-	0.495 **	−0.273 **
BFI-10 Neuroticism	0.396 **	0.371 **	0.407 **	0.495 **	-	−0.174 **
BFI-10 Conscientiousness	−0.250 **	−0.146 **	−0.152 **	−0.273 **	−0.174 **	-

Note. ** *p* < 0.01; IAT: Internet Addiction Test; BSMAS: Bergen Social Media Addiction Scale; FoMOs: Fear of Missing Out Scale; DERS: Difficulties in Emotion Regulation Scale; BFI-10: Big Five Inventory-10.

**Table 3:** Model coefficients for the serial mediation analysis (*n* = 397).

Predictor	BSMAS		FoMOs		IAT	
	β (*SE* HC0)	*p*	β (*SE* HC0)	*p*	β (*SE* HC0)	*p*
Independent variable						
DERS	0.014 (0.00)	<0.001	0.125 (0.02)	<0.001	0.068 (0.03)	0.014
BSMAS	-	-	-	-	9.197 (0.44)	<0.001
FoMOs	-	-	-	-	0.192 (0.05)	<0.001
Covariate						
BFI-10 Neuroticism	0.061 (0.01)	<0.001	0.397 (0.12)	<0.001	0.131 (0.12)	0.272
BFI-10 Conscientiousness	−0.010 (0.01)	0.455	−0.025 (0.12)	0.828	−0.395 (0.12)	0.002
*R* ^2^	0.19		0.34		0.67	
*F* HC0 (df)	34.309 (3393) ***		51.207 (4392) ***		176.752 (5391) ***	
Total effect on IAT	IAT				95% CI	
		β (*SE*)	*p*		LL	UL
DERS		0.227 (0.039)	<0.001		0.1522	0.3071
BFI-10 Neuroticism		0.807 (0.169)	<0.001		0.4752	1.1379
BFI-10 Conscientiousness		−0.496 (0.172)	0.004		−0.8352	−0.1568
*R* ^2^		0.25				
*F* HC0 (df)		52.043 (3393) ***				
Bootstrap indirect effects on IAT	IAT				95% CI	
		β (*SE*)			LL	UL
Total		0.161 (0.03)			0.0990	0.2250
BMSAS		0.128 (0.03)			0.0721	0.1858
FoMOs		0.024 (0.01)			0.0095	0.0431
BMSAS, FoMOs		0.009 (0.00)			0.0033	0.0166

Note. DERS = Difficulties in Emotion Regulation; BSMAS = Bergen Social Media Addiction Scale; FoMOs = Fear of Missing Out Scale; IAT = Internet Addiction Test. Bootstrap sample size = 5000 (two-tailed); *p* < 0.001 ***.
